# Semi-Supervised Classification of PolSAR Images Based on Co-Training of CNN and SVM with Limited Labeled Samples

**DOI:** 10.3390/s23042109

**Published:** 2023-02-13

**Authors:** Mingjun Zhao, Yinglei Cheng, Xianxiang Qin, Wangsheng Yu, Peng Wang

**Affiliations:** 1 Information and Navigation College, Air Force Engineering University, Xi’an 710077, China; 2 School of Computer Science, Northwestern Polytechnical University, Xi’an 710129, China

**Keywords:** polarimetric synthetic aperture radar (PolSAR), semi-supervised classification, co-training, convolutional neural network (CNN), residual network (ResNet), support vector machine (SVM)

## Abstract

Recently, convolutional neural networks (CNNs) have shown significant advantages in the tasks of image classification; however, these usually require a large number of labeled samples for training. In practice, it is difficult and costly to obtain sufficient labeled samples of polarimetric synthetic aperture radar (PolSAR) images. To address this problem, we propose a novel semi-supervised classification method for PolSAR images in this paper, using the co-training of CNN and a support vector machine (SVM). In our co-training method, an eight-layer CNN with residual network (ResNet) architecture is designed as the primary classifier, and an SVM is used as the auxiliary classifier. In particular, the SVM is used to enhance the performance of our algorithm in the case of limited labeled samples. In our method, more and more pseudo-labeled samples are iteratively yielded for training through a two-stage co-training of CNN and SVM, which gradually improves the performance of the two classifiers. The trained CNN is employed as the final classifier due to its strong classification capability with enough samples. We carried out experiments on two C-band airborne PolSAR images acquired by the AIRSAR systems and an L-band spaceborne PolSAR image acquired by the GaoFen-3 system. The experimental results demonstrate that the proposed method can effectively integrate the complementary advantages of SVM and CNN, providing overall classification accuracy of more than 97%, 96% and 93% with limited labeled samples (10 samples per class) for the above three images, respectively, which is superior to the state-of-the-art semi-supervised methods for PolSAR image classification.

## 1. Introduction

Polarimetric synthetic aperture radar (PolSAR) is an advanced active microwave imaging system with the strong capability of information acquisition, which can perform under all-day and all-weather conditions [1]. As an important part of PolSAR image interpretation, PolSAR image classification is valuable in both civil and military applications, and has been a hot research topic for a long time.

Traditional methods of PolSAR image classification usually focus on two aspects, i.e., feature extraction and classifier design [2]. In the aspect of feature extraction of PolSAR images, a large number of polarimetric decomposition methods have been developed successively, such as Cloude decomposition [3], Freeman decomposition [4] and Yamaguchi decomposition [5]. Besides this, some other features have also been widely used, such as the polarimetric rotation domain features [2,6], texture features [7,8,9] and color features [10]. In the aspect of classifier design, many classifiers have been developed for PolSAR image classification, such as Wishart classifier [11], decision tree [12,13], k-nearest neighbor (KNN) [14] and support vector machine (SVM) [15]. PolSAR image classification methods have been widely studied in the past decades, but due to the limited performance of the features and classifiers used, the generality and reliability of the traditional methods are still not enough to meet the needs of many practical applications.

In recent years, many deep convolutional neural networks (CNNs) have been proposed in the field of computer vision and shown powerful capabilities in many classification tasks. These CNNs have subsequently been introduced and improved in the application of PolSAR image classification [2,16,17,18,19]. Different from the traditional classification methods, CNN-based methods are not required to extract explicit features, and can integrate feature extraction and classifier design as a whole to provide superior classification results. On the other hand, however, CNNs are data-driven networks that usually require a large number of labeled samples for training. Under the condition of limited samples, the networks easily overfit the data. In practice, different to general images, PolSAR images are relatively scarce and have poor visual effects due to their special coherent imaging mechanism, which makes it difficult and costly to obtain labeled samples of PolSAR images. Therefore, it is of important practical significance to solve the problem of PolSAR image classification in the case of limited labeled samples.

From the perspective of training samples, classification methods can generally be divided into fully supervised, unsupervised and semi-supervised ones. In fully supervised classification methods, all the training samples are labeled. In contrast, in unsupervised ones, all the training samples only have the data itself, without the corresponding class labels. By comparison, in the semi-supervised methods, some training samples are labeled while the others are not, where the unlabeled samples are usually much more numerous than the labeled ones. Consequently, the semi-supervised methods can not only use the information of labeled samples, but also utilize the information of the unlabeled ones to improve the classification performance [20]. Moreover, in practice, it is feasible to obtain a larger number of unlabeled PolSAR images, which makes the semi-supervised methods more attractive for solving the problem of PolSAR image classification with limited labeled samples [14,21,22,23,24,25,26,27,28,29].

At present, there are two typical kinds of semi-supervised classification methods used for PolSAR images, including graph-based methods [21,22,24] and “pseudo labels”-based ones [14,23,26,27,29]. The semi-supervised methods based on graphs have clear mathematical theories. However, they usually involve a complicated process of graph construction, where the label propagation depends on the inversion of large matrices [20], which requires high storage overhead and is difficult to perform directly on large-scale data. In contrast, the “pseudo labels”-based methods, such as self-training [26,27], co-training [30,31] and tri-training ones [14], require low storage overhead and are widely used in PolSAR image classification. The “pseudo labels” of the unlabeled samples are their classification results, i.e., predicted labels, provided by the trained classifiers. These methods iteratively train their classifiers, which are further used to increase the amount of pseudo-labeled samples. The main difference between these “pseudo labels”-based methods is that they use different numbers of base classifiers. As their names indicate, each self-training method has a single base classifier, while the co-training and tri-training methods have two and three base classifiers, respectively. Generally speaking, the classification performance of “pseudo labels”-based methods would be improved with an increase in the number and an improvement of the performance of their base classifiers, but their complexity would increase too.

Many traditional classifiers of machine learning have been used as base classifiers in the “pseudo labels”-based classification methods, such as SVM, decision tree, adaboost and K-nearest neighbor (KNN) [23,28]. Because of their relative potential, the performance of the whole classification methods is often limited. In recent years, with the rapid development of deep learning technology, CNNs with great classification potential have been used as base classifiers of such methods, and have achieved good classification results [25,26]. However, as mentioned above, as data-driven models, CNNs depend on the sizes of labeled samples, and their performance will significantly decrease when the labeled samples are limited, even worse than the traditional classifiers.

To address these problems, in this paper, a semi-supervised classification method based on the co-training of CNN and SVM is proposed for PolSAR images. The motivations of our work are as follows. In the past few decades, SVM has been deeply studied and widely used in classification tasks. Many studies show that SVM has superior classification capabilities to many other classical classifiers, particularly with respect to small training sample sizes [32,33,34,35]. According, SVM is able to outperform CNN given limited labeled samples. Considering the superiority of CNNs with sufficient training samples, a co-training method with SVM and CNN as base classifiers is proposed so as to integrate their complementary advantages. In our method, an eight-layer CNN with a residual network (ResNet) structure [36] is designed as the primary classifier due to its excellent classification potential. Besides this, to reduce the dependence of the method on the amount of given labeled samples, SVM is introduced as an auxiliary classifier. Moreover, a two-stage co-training strategy is designed to gradually increase the amount of pseudo-labeled samples. Experiments are carried out on both airborne and spaceborne PolSAR images of different bands, and the results show that the proposed method is much superior to the state-of-the-art semi-supervised methods with very limited labeled samples.

## 2. PolSAR Image Data and Features

### 2.1. PolSAR Image Data

Each pixel of a single-look PolSAR image is usually represented by a polarimetric scattering matrix, i.e., Equation (1) [1]

(1) $$S=\left[\begin{array}{ll} S_{HH} & S_{HV}\\ S_{VH} & S_{VV} \end{array}\right]$$

where “*H*” and “*V*” denote the horizontal and vertical polarization, respectively, and $S_{kl},\;\;k,l=H,V$ is the scattering coefficient for transmitting in *l* polarization and receiving in *k* polarization. Under the reciprocity condition, the scattering matrix can be equivalent to a Pauli basis scattering vector, i.e.,

(2) $$k_{P}=\frac{1}{\sqrt{2}}{\left[S_{HH}+S_{VV},S_{HH}-S_{VV},2S_{HV}\right]}^{T}$$

where the superscript “T” indicates the transpose operation.

In order to suppress the inherent speckle noise, multi-look processing is often carried out for PolSAR images. Each pixel of the multi-look PolSAR image can be represented by a polarimetric coherence matrix [1]

(3) $$T_{3}=\langle k_{P}\cdot k_{P}^{H}\rangle =\left[\begin{array}{lll} T_{11} & T_{12} & T_{13}\\ T_{21} & T_{22} & T_{23}\\ T_{31} & T_{32} & T_{33} \end{array}\right]$$

where $T_{ij}$ is the element of the coherence matrix $T_{3}$, 〈⋅〉 denotes the ensemble averaging operation, and the superscript “H” represents the conjugate transpose operation.

### 2.2. PolSAR Image Features

In the past few decades, various polarimetric features have been developed for PolSAR images, which are in fact good representations of the original data. In this section, six polarimetric features widely used in PolSAR image classification are introduced, including three features yielded by the Cloude decomposition, the total power SPAN and two polarimetric rotation null angle features. According to Cloude decomposition [3], the conjugate symmetric polarimetric coherence matrix $T_{3}$ can be expressed as

(4) $$T_{3}=U\left[\begin{array}{lll} \lambda_{1} & 0 & 0\\ 0 & \lambda_{2} & 0\\ 0 & 0 & \lambda_{3} \end{array}\right]U^{H},\lambda_{1}\geq \lambda_{2}\geq \lambda_{3}$$

where $\lambda_{1}$, $\lambda_{2}$, and $\lambda_{3}$ are the three eigenvalues of the matrix $T_{3}$, and U is the unitary matrix composed of the three unit orthogonal eigenvectors. Thus, three features, i.e., the scattering entropy *H*, anisotropy *A* and mean alpha angle $\bar{\alpha }$, can be constructed as

(5) $$H=-\sum_{i=1}^{3}P_{i}log_{3}P_{i},\;\;A=\frac{\lambda_{2}-\lambda_{3}}{\lambda_{2}+\lambda_{3}},\;\;\bar{\alpha }=\sum_{i=1}^{3}P_{i}\alpha_{i}$$

where $P_{i}=\lambda_{i}/\left(\lambda_{1}+\lambda_{2}+\lambda_{3}\right)$ and $\alpha_{i}$ are the *i*-th eigenvector parameters. The entropy *H* represents the randomness of the scattering data, the anisotropy *A* reflects the relative influence of the second and the third eigenvalues, and the angle $\bar{\alpha }$ can be used to identify the potential average scattering mechanism of the data to a certain extent [1,3].

The total power SPAN of PolSAR images is a widely used rotation invariant feature, which equals the trace of the matrix $T_{3}$, namely, [1]

(6) $$\mathrm{SPAN}=T_{11}+T_{22}+T_{33}$$

Besides this, in recent years, the characteristics of the polarimetric rotation domain have been deeply studied, and two null angles in this domain are given by [2,6]

(7) θnull_Re[T12]=−12Angle{Re[T13]+jRe[T12]}

(8) θnull_Im[T12]=−12Angle{Im[T13]+jIm[T12]}

where Re[⋅] and Im[⋅] represent the real part and imaginary parts of the complex number, respectively, and Angle{⋅} refers to the phase of the complex number. Many studies have shown that these two features are sensitive to different terrains, and are useful for PolSAR image classification [2,18,37].

## 3. Methodology

**Figure 1:**
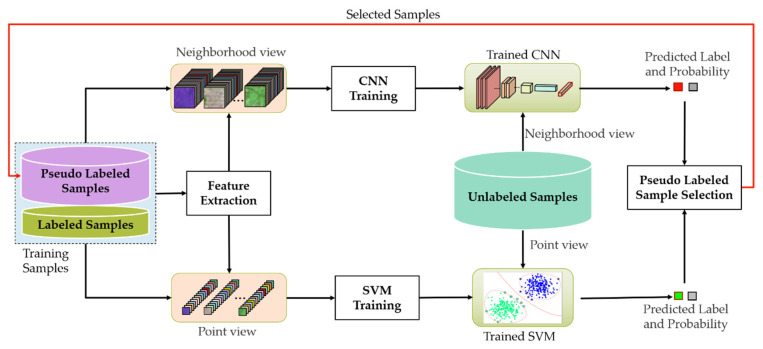
Flowchart of the proposed method.

The co-training method was originally proposed by Blum and Mitchell [38]. It usually contains two base classifiers or uses two different feature views. By taking advantage of the complementarity between different classifiers or features, the method can achieve a good performance, which cannot be achieved by using only a single classifier or a single feature view [20,30]. CNNs are able to complete the classification tasks, but they are data-driven models, and usually heavily rely on the amount of labeled training samples. When the labeled samples are limited, it is difficult to obtain satisfactory classification results using CNNs. To solve this problem, a co-training method for PolSAR image classification has been developed, combining the CNN with the SVM, which is less sensitive to the amount of labeled training samples. The flowchart of the proposed method is shown in Figure 1, and more details of the proposed method are as follows.

### 3.1. Base Classifiers

#### 3.1.1. Convolutional Neural Network

In recent years, CNN has been widely used in the field of computer vision. Especially since Krizhevsky et al. successfully used a CNN called AlexNet in the ImageNet large-scale visual recognition challenge competition in 2012 [39], CNNs have been rapidly developed. Many excellent CNNs have been proposed, such as the GoogleNet [40], VGG-Net [41], ResNet [36] and so on, the performances of which are significantly better than those of various traditional machine learning methods.

**Figure 2:**
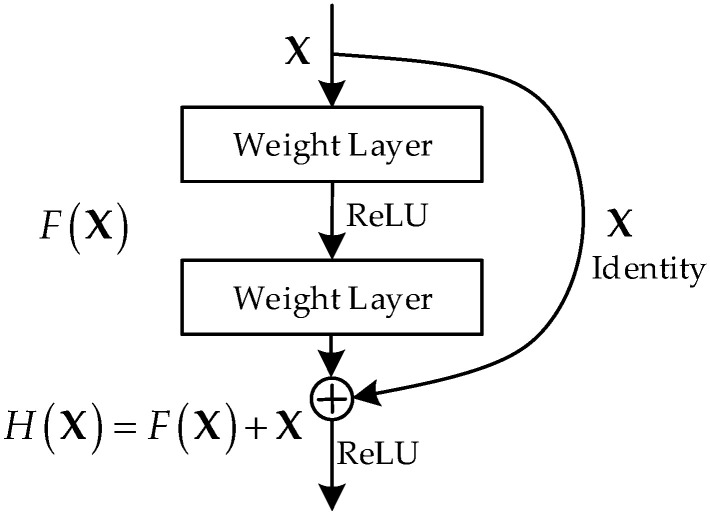
Illustration of a basic residual learning block (ResBlock) [36].

A CNN usually consists of multiple cascaded layers, including the input layer, convolutional layer, activation function, pooling layer, full connection layer, etc. With the increase in network layers, the fitting ability of the CNN often becomes stronger, while some serious problems of gradient disappearance and model degradation may occur in the model training. To address these problems, He et al. proposed a CNN named ResNet in 2015, which achieved amazing results by designing the residual learning blocks (denoted as ResBlock) [36]. The illustration of a basic ResBlock is shown in Figure 2.

In Figure 2, X denotes the input of the ResBlock, ReLU means the rectified linear unit function [42] used as a nonlinear activation function, F(X) is the mapping of multiple weight layers in the block with respect to X, and H(X) = F(X) + X is the underlying mapping of the ResBlock. Different from the conventional CNN structure, this design makes the weight layer learn the difference between H(X) and the block input X through the identity mapping formed by cross-layer connection, which is called “residual”. Many studies show that the ResBlock can effectively avoid the model degradation and gradient disappearance problems caused by the deepening of network layers, and the deep network with ResBlock has significantly better learning performance than the network with simple stacking weight layers [43].

**Figure 3:**
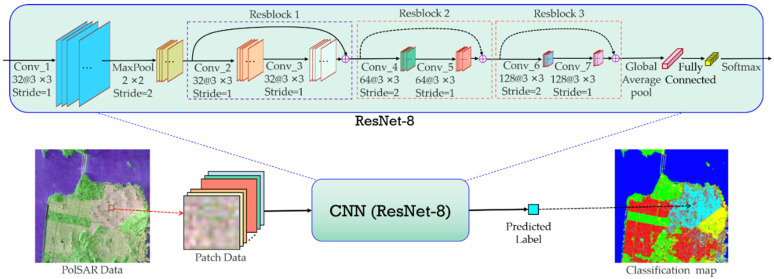
Architecture of the employed CNN and its application in PolSAR image classification.

In view of the advantages of ResBlock, a simple ResNet is designed in our co-training method for PolSAR image classification. The original ResNet with 152 layers has a strong learning ability and performs excellently on the ImageNet dataset [36]. However, since PolSAR image classification is a pixel-level task, neighborhood patches of small size are used to represent pixels, and served as the input of CNN in our method. Therefore, a CNN with many layers would cause serious losses of image features due to the convolutional and pooling operations. Accordingly, we design a shallow ResNet with eight weight layers for our task, making it more suitable for processing image patches of small sizes. The architecture of the employed CNN is shown in Figure 3.

As seen from Figure 3, this network consists of a convolutional layer, a max-pooling layer, three ResBlocks (composed of two convolutional layers), a global average pooling layer and a fully connected (FC) layer. The size of all convolutional kernels in this network is 3 × 3, and the kernel numbers in convolutional layers 1–7 are 32, 32, 32, 64, 64, 128 and 128, respectively. Besides this, the strides of convolutional layers 1–7 are 1, 1, 1, 2, 1, 2 and 1, respectively, and the stride of the max-pooling layer is 2. It also should be pointed out that a batch normalization operation is performed after each convolutional layer. Since there are eight weight layers (including seven convolutional layers and a full connection layer) in this CNN, we refer to it as ResNet-8 for simplicity in this paper. Besides this, in the classification of PolSAR images using CNN, a common approach is to represent each pixel using its neighborhood patch data, which are then classified by the trained CNN, and the predicted label is reassigned to the corresponding pixel. The main flowchart of this method is illustrated in Figure 3.

#### 3.1.2. Support Vector Machine

SVM has been deeply studied and applied in many classification tasks since the 1990s. It was one of the most widely used classifiers before deep learning was proposed, and its development is relatively mature. Many studies demonstrate that SVM has good capabilities with respect to small training sample sizes [32,33,34,35]. Considering the complementary advantages of SVM relative to CNN, we introduce SVM as the auxiliary base classifier in our co-training method.

The original SVM is proposed to solve the binary classification problem, and the data classification is completed by solving the classification hyperplane that can correctly partition the dataset and has the largest geometric interval. The main formula of SVM can be expressed as [20]

(9) $$\begin{array}{l} \min\;\frac{1}{2}w^{T}w+\gamma \sum_{i=1}^{N}\rho_{i},\gamma \geq 0\\ s.t.\;y_{i}(w^{T}x_{i}+b)\geq 1-\rho_{i}\;\\ \;\;\rho_{i}\geq 0,\;\;i=1,\ldots \ldots ,N \end{array}$$

where w and b are the normal vector and displacement term of the classification hyperplane, respectively, *N* is the number of training samples, γ is the penalty factor and $\rho_{i}$ is the relaxation variable of the *i*-th sample, which are used to improve the fault tolerance of the model.

In SVM, the problem of maximizing the geometric interval is transformed to the problem of obtaining the maximum value. Under the condition of limited samples, it still has a relatively strong learning ability, and can avoid overfitting and dimension disasters. For nonlinearly separable samples, SVM implicitly transforms them from the original data space into a high-dimensional data space via a kernel function, making the samples linearly separable in the high-dimensional space. In addition, the binary SVM can be easily extended to the multi-class SVM by using some strategies, such as the one-versus-one and one-versus-all strategies [44,45].

### 3.2. Construction of Feature Views

As a semi-supervised method based on disagreement, co-training can make full use of information by using the features of different views to improve the performance of model. According to the characteristics of CNN and SVM, two feature views of each pixel are constructed in our method. Firstly, since the inputs of a CNN are usually image patches of a certain size, each pixel is represented by its neighborhood image patch, which serves as the input of the CNN and is called the neighborhood feature view for short. Neighborhood data contain not only the information of the pixel itself, but also the spatial context information. By comparison, the data of each pixel itself are used as the input of SVM, which forms a difference view and is called point feature view here.

Each pixel of a PolSAR image can be described by a polarimetric coherence matrix, in which the non-diagonal elements are usually complex. In order to facilitate the processing of CNN and SVM, which are usually defined in the real number domain, this method separates the real and imaginary parts of the complex elements in the coherence matrix. Thus, each pixel of a PolSAR image can be represented by a nine-dimensional (9-D) real vector, namely,

(10) F1=[T11, Re[T12], Im[T12],Re[T13],Im[T13], T22, Re[T23], Im[T23], T33]T

Many studies have shown that the utilization or combination of appropriate artificially designed features can effectively alleviate the dependence of CNN on the number of training samples [2,18,37]. Therefore, the six polarimetric features described in Section 2.2 are employed as part of the input data of CNN and SVM, which can be expressed as a six-dimensional (6-D) vector as

(11) F2=[H, A, α¯, SPAN, θnull_Re[T12], θnull_Im[T12]]T

Then, by combining the 9-D original data and the 6-D polarimetric features, each pixel of a PolSAR image can be represented by a 15-dimensional (15-D) feature vector, i.e., $F=\left[F_{1};\;F_{2}\right]$. It should be pointed that, to reduce the impact of inherent speckles, PolSAR images are filtered before constructing two feature views in our method, where the method of global weighted least squares (GWLS) filtering [46] is used.

### 3.3. Co-Training Method of CNN and SVM

The co-training method has two obvious characteristics. On the one hand, unlike the fully supervised approaches that only use the labeled samples to train models, the co-training method is semi-supervised, and attempts to use the information of unlabeled samples too. On the other hand, different to traditional classification methods with a single classifier, each co-training method has two classifiers, and aims to integrate their complementary advantages. So, there are two key issues for a co-training method, i.e., how to make full use the unlabeled samples and how to integrate the two base classifiers. Focusing on these two issues, we proposed a co-training method using CNN and SVM, as shown in Algorithm 1.

There are two sample sets in our method, namely, a labeled one of small size ($L:\left\{L_{1},\;L_{2}\right\}$) and an unlabeled one of large size ($U:\left\{U_{1},\;U_{2}\right\}$). $L_{1}$ and $L_{2}$ are the labeled samples of the neighborhood view and point view, respectively. Similarly, $U_{1}$ and $U_{2}$ are the unlabeled samples of the neighborhood view and point view, respectively. As shown in Algorithm 1, the two base classifiers are trained iteratively through several learning rounds. In each round, there are four main steps, i.e., the training of base classifiers, the classification of unlabeled samples in a buffer poll, the extension of the labeled sample set via sample selection, and the updating of the unlabeled sample set and the buffer pool.

In the semi-supervised method, unlabeled samples are classified, and the samples with high reliability are selected to extend the labeled sample set, where their predicted labels, called pseudo-labels, are used. Then, the selected samples are used for training in the next learning round. With the progression of iterative learning rounds, more and more unlabeled samples become pseudo-labeled ones, which are useful for improving the performance of classifiers. In practice, there may be a large number of unlabeled samples. If all of them are classified in each learning round of the co-training method, it will be very time-consuming, and this may incur serious storage overhead. Accordingly, at the beginning of our method, a buffer pool of unlabeled samples $B:\left\{B_{1},\;B_{2}\right\}$ is constructed, and only the samples in the buffer pool are classified in each learning round.
**Algorithm 1:** Co-training of CNN and SVM**Input:**  (1)Labeled sample set $L:\left\{L_{1},\;L_{2}\right\}$;   (2)Unlabeled sample set $U:\left\{U_{1},\;U_{2}\right\}$;  (3)Classifiers: CNN, SVM;  (4)Initial parameters.*h*: initial size of buffer pool; *k*: learning round, *k* = 1; *K*: maximum learning round;$K_{1}$: maximum learning round in stage 1; M: maximum number of selected samples of each class in each learning round.**Output:**  The trained CNN and SVM **Process:** **Construct a buffer pool of unlabeled samples:** Select *h* samples randomly from $U:\left\{U_{1},\;U_{2}\right\}$ to form a buffer pool $B:\left\{B_{1},\;B_{2}\right\}$, and remove the selected samples from U. **While** k≤K
  **(1)****Training of base classifiers:** Train CNN and SVM using $L_{1}$ and $L_{2}$, respectively.  **(2)****Classification of samples in buff pool**B**:** Classify every sample $x_{i}^{\left(1\right)}$ and $x_{i}^{\left(2\right)}$ in the buffer pool $B:\left\{B_{1},\;B_{2}\right\}$ using the trained CNN and SVM, respectively.  **(3)****Extension of labeled sample set**L**by sample selection:**(a)Stage 1: when $k\leq K_{1}$, (no more than) *M* classified samples in step (2) meeting the conditions in Expression (12) are selected as pseudo-labeled samples and added to $L:\left\{L_{1},\;L_{2}\right\}$;(b)Stage 2: when $k>K_{1}$, (no more than) *M* classified samples in step (2) meeting the conditions in Expression (13) are selected as pseudo-labeled samples and added to $L:\left\{L_{1},\;L_{2}\right\}$.(c)Count the total number of selected samples, denoted as $N_{S}$.  **(4)****Update of unlabeled sample sets**U**and**B**:**(a)Remove the samples selected in step (3) from $B:\left\{B_{1},\;B_{2}\right\}$; (b)Select $2N_{S}$ new samples randomly from the unlabeled sample set $U:\left\{U_{1},\;U_{2}\right\}$ and add them to $B:\left\{B_{1},\;B_{2}\right\}$.(c)Delete the previous $2N_{S}$ selected samples from $U:\left\{U_{1},\;U_{2}\right\}$.
  **If**
U=Φ, i.e., the unlabeled sample set U is empty:   **break**  k=k+1 **End**

In each learning round of our method, the CNN and SVM are firstly trained with the samples of the neighborhood view and the point view, respectively. Then, the samples in buffer pool $B:\left\{B_{1},\;B_{2}\right\}$ are classified by the trained CNN and SVM. Next, the labeled sample set is extended by selecting some of the previously classified samples, which is the core step of our co-training method.

The classification results given by CNN are usually unreliable when only a few labeled training samples are provided, and may even be far inferior to many traditional classifiers. Consequently, in the design of this method, we divide the extension of the labeled sample set into two stages, i.e., the first *K*_1_ learning rounds of the algorithm are stage 1, and the subsequent rounds are stage 2. In stage 1, to avoid the error accumulation caused by the unreliable initial classification performed by CNN, the classification results yielded by SVM are taken more seriously. The condition of selecting the training samples in this stage can be expressed as

(12) $$\left\{X_{1},X_{2}\right\}=\left\{\left\{x_{i}^{\left(1\right)},x_{i}^{\left(2\right)}\right\}\in B|\begin{array}{l} C_{\mathrm{CNN}}\left(x_{i}^{\left(1\right)}\right)=C_{\mathrm{SVM}}\left(x_{i}^{\left(2\right)}\right),\\ P_{\mathrm{SVM}}\left(x_{i}^{\left(2\right)}\right)>Th_{p} \end{array}\right\}$$

where $X_{1}$ and $X_{2}$ are the selected sample sets of the neighborhood view and the point view, respectively, $\left\{x_{i}^{\left(1\right)},x_{i}^{\left(2\right)}\right\}\in B$ is the *i*-th sample $x_{i}$ with two feature views in buffer pool B, $C_{\mathrm{CNN}}\left(x_{i}^{\left(1\right)}\right)$ and $C_{\mathrm{SVM}}\left(x_{i}^{\left(2\right)}\right)$ are the labels of $x_{i}$ predicted by CNN and SVM, respectively, $P_{\mathrm{SVM}}\left(x_{i}^{\left(2\right)}\right)$ is the prediction probability corresponding to $C_{\mathrm{SVM}}\left(x_{i}^{\left(2\right)}\right)$, and $Th_{p}$ is the threshold of prediction probability, which is set as 50% in this paper. This means that stage 1 selects the samples whose prediction labels given by the two classifiers are the same, and the prediction probability yielded by SVM is greater than 50%. The prediction probability yielded by CNN is not considered due to its relative unreliability. Besides this, the prediction labels of the selected samples are used as their “pseudo labels”.

After several learning rounds in stage 1, the classification results of both CNN and SVM become more credible, and are then combined to select samples. So, in stage 2, the prediction labels and probabilities of the two classifiers are all considered, and the condition is given as

(13) $$\left\{X_{1},X_{2}\right\}=\left\{\left\{x_{i}^{\left(1\right)},x_{i}^{\left(2\right)}\right\}\in B|\begin{array}{l} C_{\mathrm{CNN}}\left(x_{i}^{\left(1\right)}\right)=C_{\mathrm{SVM}}\left(x_{i}^{\left(2\right)}\right),\\ P_{\mathrm{CNN}}\left(x_{i}^{\left(1\right)}\right)>Th_{p}\;\;\mathrm{or}\;\;P_{\mathrm{SVM}}\left(x_{i}^{\left(2\right)}\right)>Th_{p} \end{array}\right\}$$

where $P_{\mathrm{CNN}}\left(x_{i}^{\left(1\right)}\right)$ is the prediction probability of sample $x_{i}$ given by CNN. This means that the method selects the samples whose labels predicted by the two classifiers are the same, and the prediction probability of any classifier is greater than $Th_{p}$, i.e., 50%. Compared to condition 1, it is more relaxed, and allows more samples to be selected.

Then, in the last step of each learning round, the unlabeled sample set and the buffer pool are updated. The previously selected samples are deleted from the buffer pool, and some new samples are randomly selected and removed from set U, which are then added into the buffer pool B. The size of U decreases while the size of B increases, since the number of selected samples is twice the total number of “pseudo-labeled” samples selected in the current learning round.

The algorithm will stop if all the samples of the initial unlabeled sample set are labeled, or the learning round reaches the given maximum number. Thus, two trained classifiers, i.e., CNN and SVM, are obtained via the co-training procedure. Since CNN often achieves superior classification performance with sufficient samples, this algorithm uses the trained CNN as the final classifier. This is also the reason we call the CNN a primary classifier in our co-training method.

## 4. Experimental Results and Discussions

### 4.1. Datasets Description and Parameters Settings

**Figure 4:**
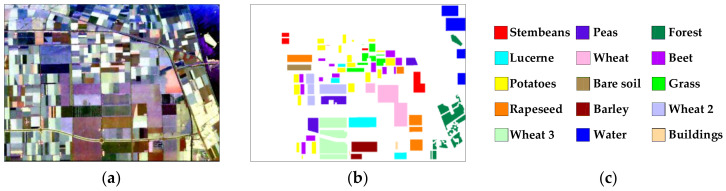
(**a**) Pauli-RGB image of dataset 1, (**b**) ground-truth [17] and (**c**) legend.

To evaluate the performance of the proposed method, three actual datasets of PolSAR images are used in the experiments in this paper. Dataset 1, acquired by the AIRSAR system in 1989, is an L-band PolSAR image of a region in Flevoland, Netherlands, with the image size of 750 × 1024 pixels. Its Pauli-RGB image, ground-truth map and legend are shown in Figure 4a–c, respectively. It has 15 labeled terrain categories, including stembeans, peas, forest, lucerne, wheat, beet, potatoes, bare soil, grass, rapeseed, barley, wheat 2, wheat 3, water, and buildings. The total number of labeled pixels is 157,296.

**Figure 5:**
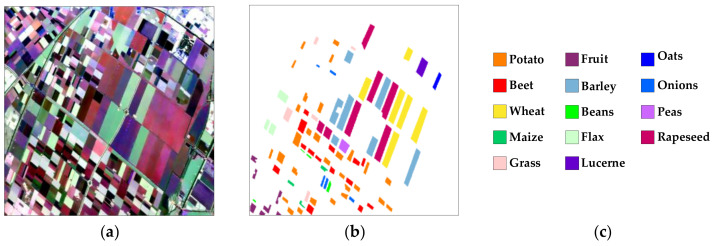
(**a**) Pauli-RGB image of dataset 2, (**b**) ground-truth [17] and (**c**) legend.

Dataset 2, acquired by the AIRSAR system in 1991, is an L-band PolSAR image of another region in Flevoland, Netherlands, consisting of 1020 × 1024 pixels. Its Pauli-RGB image, ground-truth map and legend are shown in Figure 5a–c, respectively. It has 14 labeled terrain categories, i.e., potato, fruit, oats, beet, barley, onions, wheat, beans, peas, maize, flax, rapeseed, grass, and lucerne. The total number of labeled pixels is 135,350.

**Figure 6:**
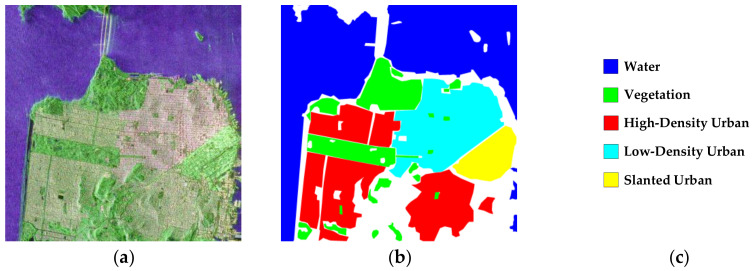
(**a**) Pauli-RGB image of dataset 3, (**b**) ground-truth [47] and (**c**) legend.

Dataset 3, acquired by the GaoFen-3 system in 2018, is a C-band PolSAR image of a region in San Francisco, USA, with the image size of 2000 × 2000 pixels. Its Pauli-RGB image, ground-truth map and legend are shown in Figure 6a–c, respectively. It has five labeled terrain classes, including water, vegetation, high-density urban, low-density urban and inclined urban areas. The total number of labeled pixels is 3,136,780.

In our experiment, the ResNet-8 given in Section 3.1.1 is employed as the CNN. The size of image patches of the neighborhood view is 15 × 15 pixels. For training CNN, the stochastic gradient descent (SGD) method is adopted, Adam with a learning rate of 0.01 is used as the optimizer, and the cross entropy loss function is used. Besides this, the SVM with the radial basis function (RBF) kernel is used, which is implemented by the SVC function in the *sklearn* package [48], and its parameters are set by the grid searching algorithm using the *GridSearchCV* function. The maximum learning round *K* = 15, the maximum learning round of stage 1 is set as $K_{1}=4$, the maximum number of selected samples per class in each learning round is set as *M* = 20, and the initial sizes of buffer pools are set as *h* = 3000, 5000, and 6000 for datasets 1–3, respectively. In addition, to avoid too many unlabeled samples occupying the memory, a certain proportion of samples indicated by the ground-truth map are randomly selected as unlabeled samples. These proportions are set as 5%, 10% and 0.5% for datasets 1–3, respectively.

### 4.2. Comparison of Fully Supervised SVM and CNN

**Figure 7:**
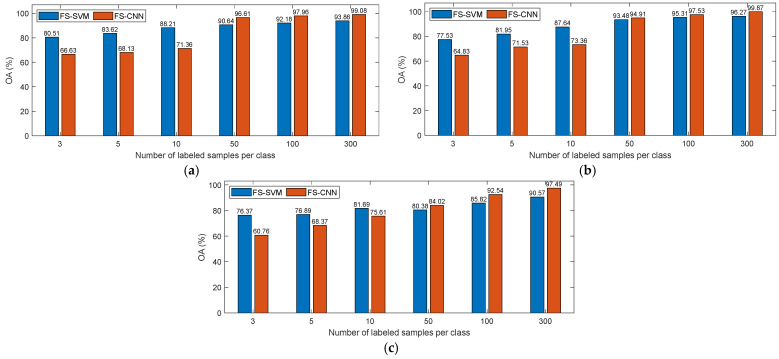
Classification OA values obtained by FS-SVM and FS-CNN with different labeled sample sizes on (**a**) dataset 1, (**b**) dataset 2 and (**c**) dataset 3.

One important motivation of our proposed method is that the SVM and CNN generally have complementary classification advantages when given different amounts of labeled training samples. To further validate this conclusion, in this section, the two classifiers are applied to the previous three datasets of PolSAR images given different numbers of labeled samples. Since the classifiers are trained in a fully supervised manner, they are called fully supervised SVM (FS-SVM) and fully supervised CNN (FS-CNN), respectively. In the experiments, 3, 5, 10, 50, 100 and 300 labeled samples per class (LSPC) are randomly selected as training samples for each dataset. Then, the PolSAR images are classified using the trained classifiers, and the overall accuracy (OA) values of classification results are calculated. Figure 7a–c show the OA values obtained with different numbers of LSPC for datasets 1–3, respectively.

As can be observed in Figure 7, with the increase in the sample size, the OA value obtained by CNN or SVM increases as well, which means that the classification performance of the two classifiers is positively related to the sample size. In addition, according to Figure 7a–c, when the number of LSPC does not exceed 10, the OA value obtained by SVM is significantly higher than that obtained by CNN, regardless of whether we use dataset 1, 2 or 3. It can be seen that in the case of limited labeled samples, the SVM achieves a better classification performance than the CNN, and the classification results yielded by SVM are more reliable. However, with the increase in sample size, the increase in OA value given by SVM is relatively small, while that given by CNN is relatively large. When the number of samples exceeds 50 for each category, the OA values given by CNN exceed those given by SVM. This indicates that the CNN has a stronger classification ability than the SVM when there is a large number of samples. To sum up, SVM and CNN show different classification advantages with different numbers of labeled samples, which supports the utility of the proposed co-training method using these two classifiers.

### 4.3. Comparison of Co-Training and Self-Training Methods

**Figure 8:**
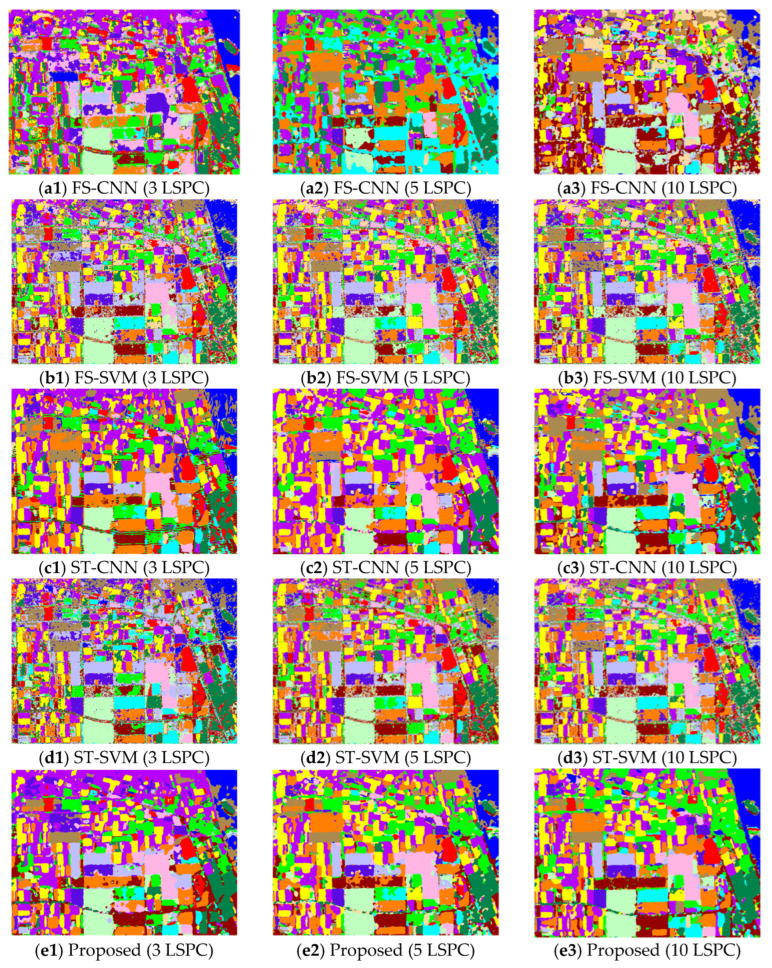
Classification maps of dataset 1 given by five methods with different sample sizes. The maps from up to down are given by FS-CNN, FS-SVM, ST-CNN, ST-SVM and the proposed methods, respectively, and those from left to right have 3, 5, and 10 LSPCs, respectively.

**Figure 9:**
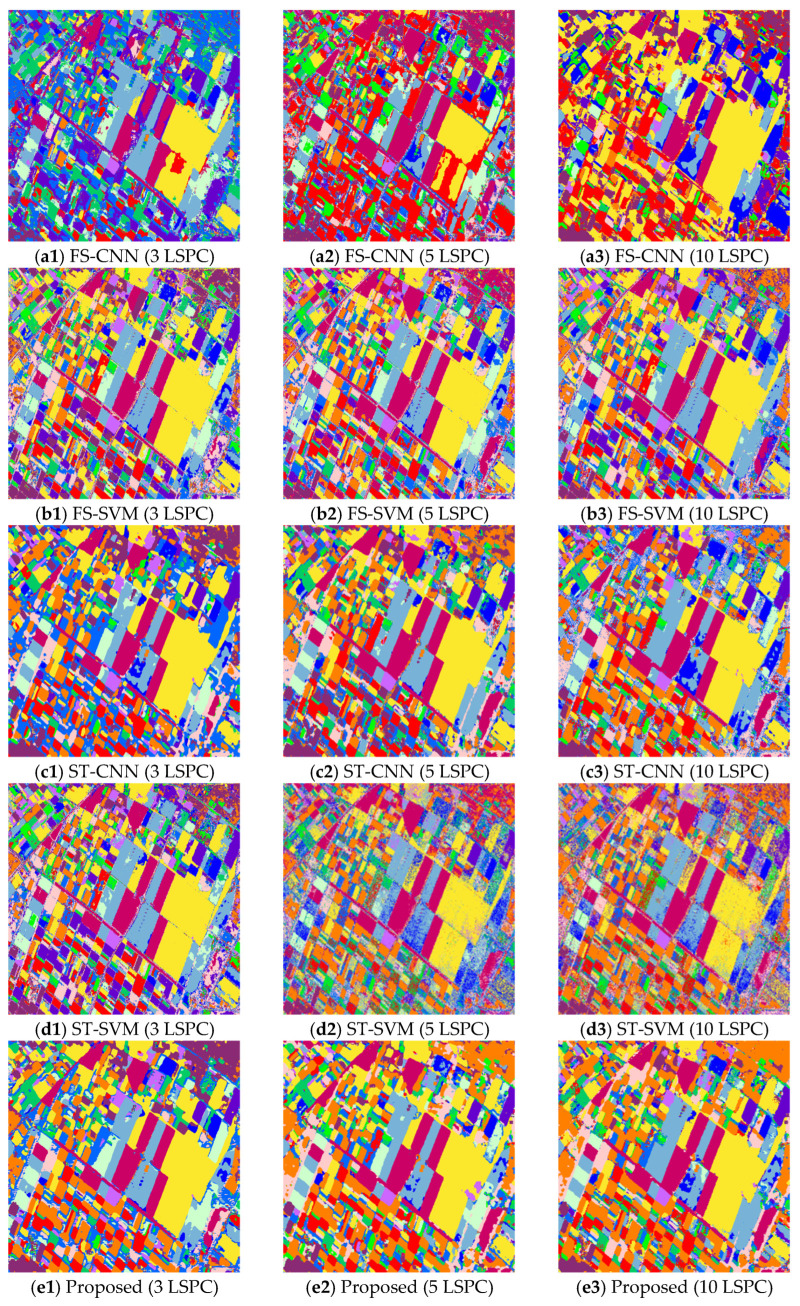
Classification maps of dataset 2 given by the five methods with different sample sizes. The maps from up to down are given by FS-CNN, FS-SVM, ST-CNN, ST-SVM and the proposed methods, respectively, and those from left to right have 3, 5, and 10 LSPCs, respectively.

**Figure 10:**
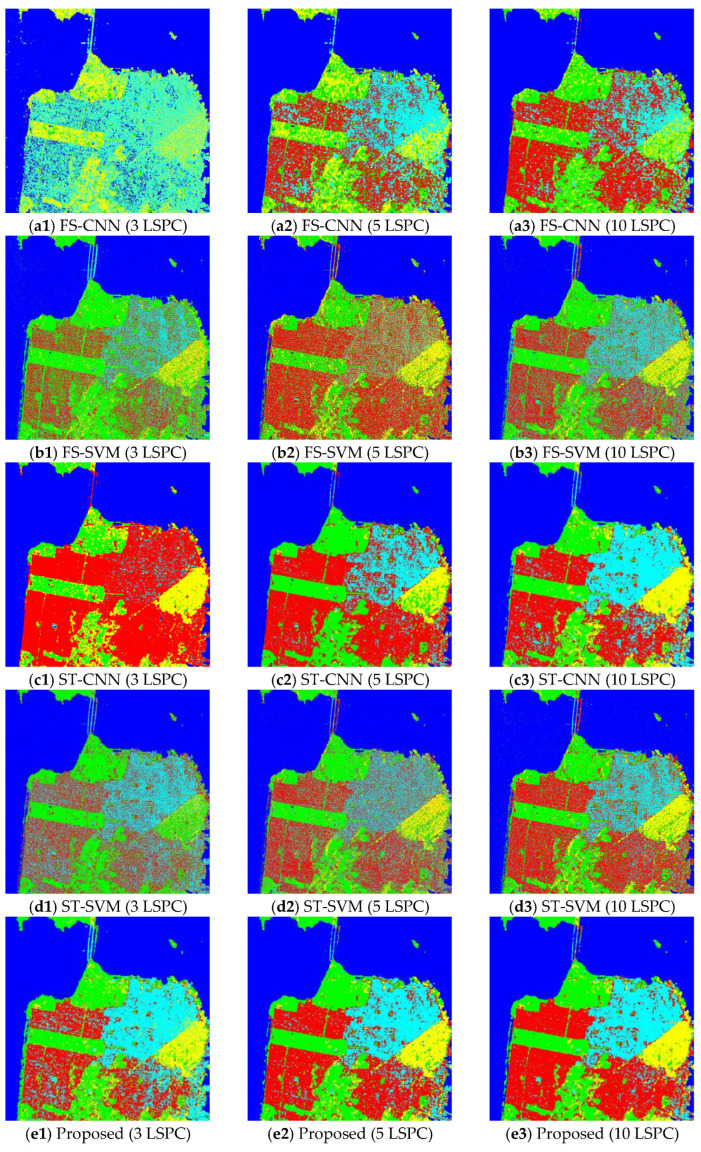
Classification maps of dataset 3 given by five methods with different sample sizes. The maps from up to down are given by FS-CNN, FS-SVM, ST-CNN, ST-SVM and the proposed methods, respectively, and those from left to right have 3, 5, and 10 LSPCs, respectively.

In this section, to evaluate the performance of the proposed method, it is applied to the three PolSAR image datasets with limited numbers of labeled samples. The experiments are carried out under the conditions of 3, 5 and 10 LSPCs. Two fully supervised methods (FS-CNN and FS-SVM) and two self-training methods (i.e., the self-training CNN (ST-CNN) and self-training SVM (ST-SVM)) are used for comparison. For fair comparison, the classifiers used in the compared methods are consistent with those used in the co-training method. The classification maps of datasets 1–3 obtained by the five different methods with different sizes of labeled training samples are shown in Figure 8, Figure 9 and Figure 10, respectively. Then, the corresponding values of the OA and Kappa coefficient of the classification results are calculated and listed in Table 1.

By comparing the classification maps in Figure 8a1–a3, Figure 9a1–a3 and Figure 10a1–a3 and the corresponding ground-truth maps in Figure 4, Figure 5 and Figure 6, it can be observed that poor classification results are obtained by the FS-CNN when only a few training samples are provided. A large number of pixels are misclassified, especially under the condition of 3 LSPCs, such as those of peas and wheat, wheat 2 and rapeseed for dataset 1, those of potato, beet and fruit for dataset 2, and those of vegetation and high-density urban for dataset 3. So, the corresponding values of OA and Kappa coefficients of the classification results are small, as shown in Table 1. By comparison, the FS-SVM yields much better classification results than the FS-CNN, i.e., the classification maps shown in Figure 8b1–b3, Figure 9b1–b3 and Figure 10b1–b3 are more similar to the corresponding ground-truth maps, and the OA values and Kappa coefficients are much higher. These results have further validated the superiority of SVM over CNN with very limited training samples.

By comparing the classification maps in Figure 8a1–a3, Figure 9a1–a3, Figure 10a1–a3, Figure 8c1–c3, Figure 9c1–c3 and Figure 10c1–c3, we can see that the semi-supervised ST-CNN obtained significantly better classification results than the FS-CNN under the same conditions. For example, the obtained OA values increased about 20% for dataset 1, i.e., from 66.63% to 85.07% with 3 LSPCs, from 68.13% to 88.51% with 5 LSPCs, and from 71.36% to 90.48% with 10 LSPCs. Similar results can be observed for the Kappa coefficients. This is mainly because many unlabeled samples were effectively utilized for training the classifiers. In contrast, though the semi-supervised ST-SVM obtained better results than the FS-SVM under the same conditions, the improvement is relatively small. The classification maps, OA values and Kappa coefficients by the FS-SVM and ST-SVM are similar. For example, the OA values given by ST-SVM are only about 1.5% more than those given by FS-SVM. These results indicate that the SVM is less sensitive to the number of samples than the CNN.

Compared with the previous four compared methods, the proposed co-training method provided the best classification results under the same conditions, i.e., the classification maps shown in Figure 8e1–e3, Figure 9e1–e3 and Figure 10e1–e3 are most similar to the ground-truth, and the obtained values of OA and Kappa coefficients are the highest. For example, as shown in Table 1, the OA value of dataset 1 given by the proposed method with 10 LSPCs is 97.84%, which is much greater than the 90.48% and 89.69% given by the ST-CNN and ST-SVM, respectively. Similar results can be observed for other datasets with different numbers of LSPC. These results reflect the positive role of SVM in our co-training method. Under the condition of 10 LSPCs, the OA values yielded by the SVM were more than 80% for these datasets, which ensures the reliability of most “pseudo-labeled” samples selected in the initial learning rounds of the method. It is worth noting that, even when very limited samples were provided, i.e., three LSPCs, acceptable classification results were still obtained by the proposed co-training method—about 90% for these datasets, which value is much better than the compared self-training ones. These results indicate that our co-training method can effectively integrate the advantages of CNN and SVM.

**Figure 11:**
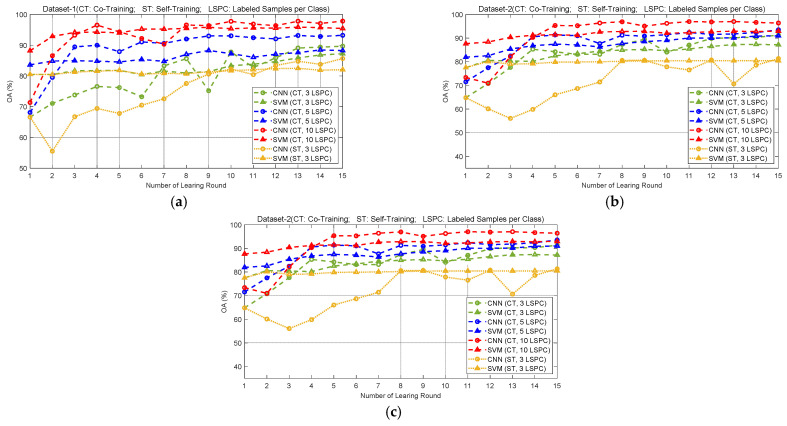
OA values obtained by SVM and CNN in different learning rounds of co-training and self-training methods applied to (**a**) dataset 1, (**b**) dataset 2 and (**c**) dataset 3, where “CT” denotes “Co-Training”, “ST” denotes “Self-Training” and “LSPC” denotes “Labeled Samples per Class”.

In order to further analyze the roles of different base classifiers in our method, the OA values obtained by SVM and CNN are also calculated in different learning rounds of the co-training procedure under the conditions of 3, 5 and 10 LSPCs. The obtained OA curves of datasets 1–3 are shown by the dashed lines in Figure 11a–c, respectively. Moreover, the obtained OA curves in different learning rounds of the ST-SVM and ST-CNN methods under the condition of three LSPCs are also presented, which are shown in Figure 11 using the orange dotted lines with triangle and circle markers, respectively.

By comparing the six OA curves (dashed lines) of each dataset given by the base classifiers in the co-training procedures, some important results are given, which can be summarized as follows.

(1) For any base classifier, SVM or CNN, the obtained OA curve of each dataset is higher when more initial labeled samples are provided. That is, the red curves (10 LSPC) are higher than the blue ones (5 LSPC), and the green ones ((3 LSPC)) are the lowest. This indicates that the initial labeled sample size has a significant impact on the performance of the classifiers.

(2) In the first learning rounds, since there is no pseudo-labeled sample, the two classifiers conduct full supervised learning with the given labeled samples, and the obtained OA values of all datasets are small, while those obtained by SVM are obviously greater than those given by CNN. These results are consistent with those shown in Figure 8a1–b3, Figure 9a1–b3 and Figure 10a1–b3. With the increase in the number of learning rounds, the OA values obtained by each base classifier increase in the overall trend. This indicates that the performance of the two classifiers, especially the CNN, can be effectively improved by using more pseudo-labeled samples for training.

(3) As the number of learning rounds increases, the OA values obtained by CNN increase faster than those given by SVM. After a certain number of learning rounds, the OA values obtained by CNN are almost aways greater than those obtained by SVM. This is also the reason why the CNN is used as the primary classifier of our co-training method, and is used for the final classification. This result further validates that CNN is superior to SVM for classification when a large number of samples are provided.

In addition, by comparing the OA curves given by the same classifier (CNN or SVM) but in different training approaches, some important results are derived.

(1) As shown by the green and orange curves with circle markers in Figure 11, for each dataset, the CNN trained by co-training provides significantly greater OA values than the same CNN trained by self-training, even though they yield the same results in the first learning round. This indicates that the SVM is useful for improving the performance of the trained CNN in our co-training method.

(2) Similarly, as shown by the green and orange curves with triangle markers in Figure 11, the SVM trained by co-training also provides greater OA values than the same SVM trained by self-training, though the improvement is not as obvious as that given by CNN. It indicates that the CNN is also useful for improving the performance of the SVM trained by co-training.

These results demonstrate that the two base classifiers promote each other well following co-training. In summary, compared to the self-training methods, the co-training method can make better use of the unlabeled samples and yield superior classifiers.

In order to further evaluate the effectiveness of the SVM used in the proposed method, two other traditional classifiers, i.e., K-nearest neighbor (KNN) and multi-layer perceptron (MLP), are used as auxiliary classifiers and replace SVM in our co-training method for comparison; the corresponding methods are briefly denoted as CNN-KNN and CNN-MLP, respectively. In the experiment, the two classifiers are implemented using the functions in the *sklearn* package [48]. The MLP contains two hidden layers, of which the neuron numbers are 20 and 30, respectively. Besides this, the parameters of KNN and the other parameters of MLP are the default in the package. The two methods are applied to the previous three datasets under the conditions of 3, 5 and 10 LSPCs, and the obtained values of the OA and Kappa coefficient are listed in Table 1. Considering the length of the paper, the corresponding classification maps are not given.

As can be seen in Table 1, under the same limited sample conditions, when KNN or MLP is used to replace SVM as the auxiliary classifier in the framework of our co-training method, the obtained values of the OA and Kappa coefficient are both higher than those obtained by the FS-CNN. For example, when the given sample number is three LSPCs, the OA values of CNN-KNN and CNN-MLP for dataset 1 are 81.04% and 86.09%, respectively, which are significantly higher than the 66.63% of FS-CNN. Similar results can be obtained for the two other sample conditions and for the other two datasets. These results demonstrate that these traditional classifiers are also helpful in promoting the performance of the trained CNN. However, in comparison, the OA and Kappa coefficients obtained by our co-training method using SVM are the highest, which verifies that SVM is superior to KNN and MLP under the condition of limited samples, and is also more suitable for use in cooperation with CNN.

### 4.4. Comparison with Other Semi-Supervised Methods

In order to further evaluate the performance of the proposed method, many other state-of-the-art semi-supervised classification methods for PolSAR images with limited labeled samples are compared in this section. The methods used for comparison include tri-training with neighborhood minimum spanning tree (TT-NMST) [14], self-training with neighborhood minimum spanning tree (ST-NMST) [29], stacked sparse auto-encoder (SSAE) [49], two recurrent complex-valued CNNs (RCV-CNN1 and RCV-CNN2) [25], the superpixel restrained deep neural network with multiple decisions (SRDNN-MD) [22], the superpixel graph-based CNN (SPGraphCNN) [21], and two methods based on a spatial anchor graph (SSA1 and SSA2) [24].

**Figure 12:**
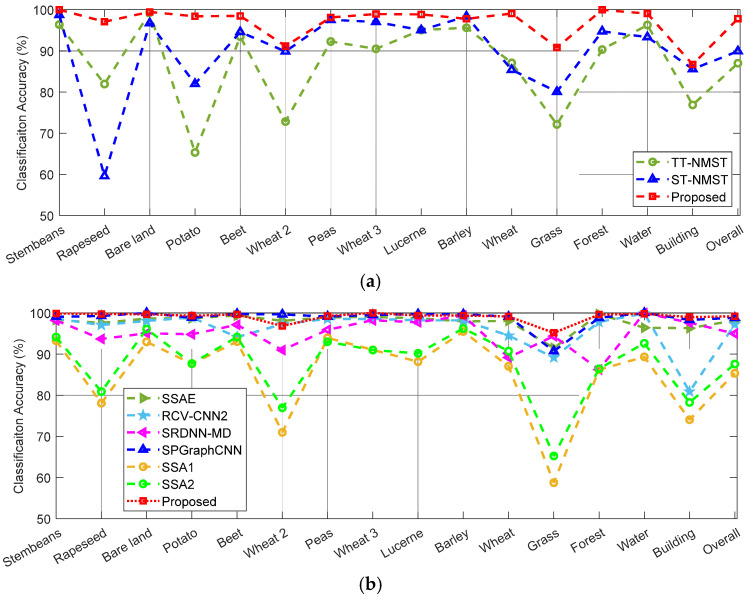

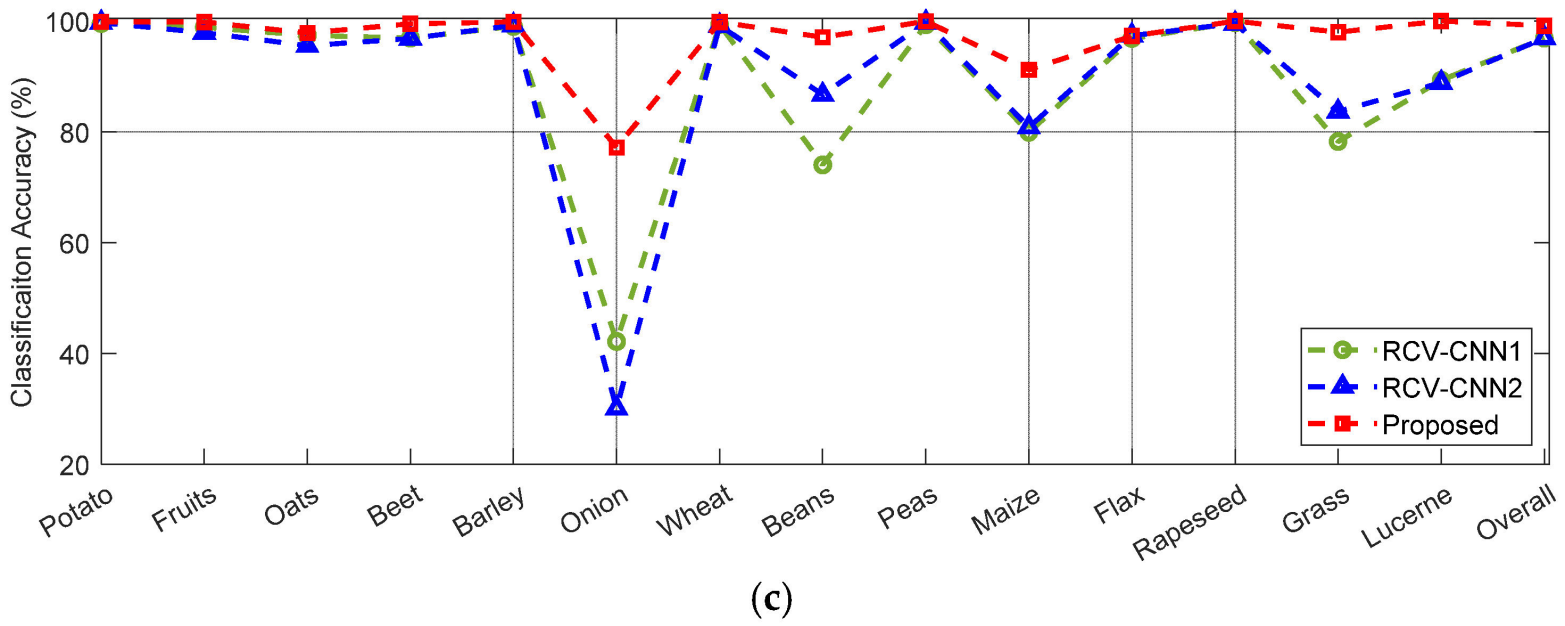
Classification accuracy values obtained by different methods, corresponding to those given in (**a**) Table 2, (**b**) Table 3 and (**c**) Table 4.

Note that, in many studies of PolSAR image classification, datasets 1–2 are commonly used for method comparison, and these are used here. Moreover, in most existing papers on PolSAR image classification with limited samples, there are two typical approaches to tingset the amount of labeled training samples. One is to select 10 LSPCs, as done in the previous section, and the other is to select a certain ratio, such as 1% of labeled samples, for training. Therefore, in this section, we compare different methods separately according to these two methods. It should be pointed out that not all of the existing methods mentioned above have been employed on both datasets 1 and 2, or used two methods to select the samples, so only the tested results given in the literature are used for comparison. The accuracy of each category, as well as the OA and Kappa coefficients of datasets 1–2, obtained by different methods with different amounts of training samples, are presented in Table 2, Table 3 and Table 4. Besides this, in order to visually compare these results, the classification accuracy values listed in Table 2, Table 3 and Table 4 are also shown graphically in Figure 12a–c, respectively.

As shown in Table 2 and Figure 12a, for the classification of dataset 1 under the condition of 10 LSPCs, the proposed method obtains the highest classification accuracy for almost all categories. Consequently, the OA value given by our method is as high as 97.84%, which is significantly greater than those given by the two methods used for comparison, i.e., 87.01% by TT-NMST and 89.92% by ST-NMST. Similarly, the Kappa coefficient given by our method is 0.9764, which is much greater than the 0.8542 and 0.8852 given by the other two methods.

Besides this, as shown in Table 3 and Figure 12b, for the classification of dataset 1 under the condition of 1% labeled samples, the SPGraphCNN and our method provide better results than the other five methods used for comparison. The highest accuracy for most categories is obtained by these two methods, i.e., among the 15 categories, the SPGraphCNN yields the highest accuracy for 6 categories, while our method is the best for the other 8 categories. The OA given by SPGraphCNN is 98.82%, which is obviously larger than that given by the other five methods used for comparison. By comparison, our method provides the highest OA—99.20%—and the largest Kappa coefficient—0.9913, which are somewhat better than those given by SPGraphCNN.

In addition, as shown in Table 4 and Figure 12c, for the classification of dataset 2 under the condition of 1% labeled samples, the proposed method also obtains the highest classification accuracy for most categories. The OA value given by our method is 99.17%, which is obviously greater than those of the two methods used for comparison, i.e., 96.93% given by RCV-CNN1 and 96.97% given by RCV-CNN2. Similarly, our method gives the largest Kappa coefficient, 0. 9903, compared to the other two methods.

From the above results, it can be concluded that the proposed co-training method can address the problem of PolSAR image classification with limited labeled samples very well, and has obvious advantages over the state-of-the-art semi-supervised classification approach used for PolSAR images.

## 5. Conclusions

In this paper, to improve PolSAR image classification with limited labeled samples, a novel semi-supervised classification method has been proposed that integrates the complementary advantages of CNN and SVM in a co-training framework. In our method, there are two base classifiers, i.e., an eight-layer CNN with a ResNet architecture and an SVM with a radial basis function (RBF) kernel. It has been shown that the two base classifiers can promote each other very well in the co-training framework, making the method much more powerful and able to address the problem of limited labeled samples. We performed many experiments on the L-band and C-band PolSAR image datasets acquired by the AIRSAR and GaoFen-3 systems. The experimental results demonstrate that the proposed method can effectively integrate the complementary advantages of SVM and CNN, providing overall classification accuracies of more than 97%, 96% and 93% with limited labeled samples (10 samples per class) for the above three images, respectively, which values are superior to those of the self-training SVM, the self-training CNN and the other state-of-the-art semi-supervised classification methods used for PolSAR images when few labeled samples are provided.

It should be noted that the framework of our co-training method is not limited to PolSAR image classification, but expands to solving the problem of image classification under the condition of limited labeled samples. It can be predicted that, for the classification of other images, our method is also theoretically applicable if the labeled samples are limited, but the results will be slightly different in feature extraction. In the future, we will carry out more experiments and analyses on our method, applying it in other image classification tasks, such as hyperspectral and infrared image classification.

## Figures and Tables

**Table 1:** OA (%) and Kappa coefficients of datasets 1–3 given by seven methods.

Dataset	Method	OA (%)			Kappa		
		3 LSPC	5 LSPC	10 LSPC	3 LSPC	5 LSPC	10 LSPC
Dataset 1	FS-CNN	66.63	68.13	71.36	0.6364	0.6343	0.6837
	FS-SVM	80.51	83.62	88.21	0.7883	0.8221	0.8717
	ST-CNN	85.07	88.51	90.48	0.8377	0.8766	0.8963
	ST-SVM	82.03	85.01	89.69	0.8026	0.8366	0.8875
	CNN-KNN	81.04	89.31	92.29	0.7941	0.8839	0.9160
	CNN-MLP	86.09	91.14	93.58	0.8486	0.9033	0.9299
	Proposed	**89.68**	**93.22**	**97.84**	**0.8759**	**0.9261**	**0.9764**
Dataset 2	FS-CNN	64.83	71.53	73.36	0.6031	0.6754	0.6920
	FS-SVM	77.53	81.95	87.64	0.7435	0.7894	0.8311
	ST-CNN	83.79	86.45	94.69	0.8158	0.8430	0.9379
	ST-SVM	80.46	83.58	89.73	0.7816	0.8094	0.8801
	CNN-KNN	76.89	88.22	92.15	0.7372	0.8632	0.9084
	CNN-MLP	82.02	91.44	94.86	0.7932	0.9001	0.9398
	Proposed	**91.28**	**93.52**	**96.38**	**0.8984**	**0.9241**	**0.9576**
Dataset 3	FS-CNN	60.76	68.37	75.61	0.4521	0.6139	0.7076
	FS-SVM	76.37	76.89	81.69	0.6678	0.6715	0.7402
	ST-CNN	81.53	90.15	91.83	0.7322	0.8588	0.8839
	ST-SVM	78.10	80.39	84.63	0.6874	0.7201	0.7813
	CNN-KNN	75.91	85.83	86.45	0.6633	0.7988	0.8056
	CNN-MLP	80.51	84.30	87.23	0.7206	0.7757	0.8164
	Proposed	**87.75**	**92.21**	**93.97**	**0.8269**	**0.8890**	**0.9140**

Bold font represents the highest value of each column of classification results.

**Table 2:** Accuracy and Kappa coefficients of dataset 1 for three semi-supervised methods under the condition of 10 LSPCs.

Method.	Stembeans	Rapeseed	Bare Land	Potato	Beet	Wheat 2	Peas	Wheat 3	Lucerne
**TT-NMST [14]**	96.40	81.95	99.31	65.31	93.45	72.84	92.29	90.50	95.07
**ST-NMST [29]**	98.75	59.58	96.75	81.99	94.60	89.86	97.56	97.05	95.06
**Proposed**	**100**	**97.10**	**99.48**	**98.41**	**98.51**	**91.17**	**98.14**	**98.96**	**98.86**
**Method**	Barley	Wheat	Grass	Forest	Water	Building	OA	Kappa	
**TT-NMST [14]**	95.64	87.09	72.13	90.32	96.30	76.87	87.01	0.8542	
**ST-NMST [29]**	**98.39**	85.41	80.08	94.77	93.35	85.58	89.92	0.8852	
**Proposed**	97.80	**99.08**	**90.83**	**99.98**	**99.08**	**86.70**	**97.84**	**0.9764**	

Bold font represents the highest value of each column of classification results.

**Table 3:** Accuracy and Kappa coefficients of dataset 1 for seven semi-supervised methods under the condition of 1% sample ratio.

Method	Stembeans	Rapeseed	Bare Land	Potato	Beet	Wheat 2	Peas	Wheat 3	Lucerne
**SSAE** [49]	98.33	97.57	98.66	98.72	99.40	98.10	98.97	99.00	98.72
**RCV-CNN2** [25]	98.61	97.07	98.05	98.90	94.14	97.28	98.56	98.56	98.22
**SRDNN-MD** [22]	98.18	93.68	95.06	94.81	97.13	90.98	95.91	98.23	97.72
**SPGraphCNN** [21]	99.07	99.26	**100**	98.83	**99.76**	**99.65**	99.12	99.50	**99.72**
**SSA1** [24]	93.25	78.08	92.99	87.64	93.05	70.98	93.95	91.03	88.15
**SSA2** [24]	94.14	80.88	96.09	87.71	94.17	76.96	93.01	90.98	90.19
**Proposed**	**99.87**	**99.77**	99.68	**99.35**	99.63	96.84	**99.26**	**100**	99.38
**Method**	Barley	Wheat	Grass	Forest	Water	Building	OA	Kappa	
**SSAE** [49]	97.93	98.08	91.50	**99.71**	96.41	96.31	98.18	0.9802	
**RCV-CNN2** [25]	98.20	94.50	89.17	97.81	99.89	80.88	97.22	0.8930	
**SRDNN-MD** [22]	99.38	89.17	94.37	86.24	**100**	97.64	94.98	0.9453	
**SPGraphCNN** [21]	**99.68**	99.10	90.72	98.81	**100**	98.31	98.82	NULL	
**SSA1** [24]	95.50	87.04	58.75	86.20	89.33	74.06	85.33	NULL	
**SSA2** [24]	96.23	90.74	65.25	86.44	92.63	78.25	87.58	NULL	
**Proposed**	99.38	**99.16**	**95.17**	99.61	99.81	**99.00**	**99.20**	**0.9913**	

NULL means that the value is not provided in the literature. Bold font represents the highest value of each column of classification results.

**Table 4:** Accuracy and Kappa coefficients of dataset 2 for three semi-supervised methods under the condition of 1% sample ratio.

Method	Potato	Fruits	Oats	Beet	Barley	Onion	Wheat	Beans
**RCV-CNN1** [25]	99.56	98.87	97.42	96.92	99.03	42.16	99.38	74.03
**RCV-CNN2** [25]	99.71	97.91	95.55	96.86	99.23	30.09	99.20	86.69
**Proposed**	**99.87**	**99.84**	**97.85**	**99.52**	**99.86**	**77.22**	**99.81**	**97.08**
**Method**	Peas	Maize	Flax	Rapeseed	Grass	Lucerne	OA	Kappa
**RCV-CNN1** [25]	99.35	79.92	96.88	99.71	78.26	89.33	96.93	0.8852
**RCV-CNN2** [25]	99.77	80.85	**97.40**	99.55	83.68	88.89	96.97	0.8888
**Proposed**	**99.95**	**91.21**	97.31	**99.99**	**98.01**	**100**	**99.17**	**0.9903**

Bold font represents the highest value of each column of classification results.

## Data Availability

Not applicable.
